# Exploring Healthcare Experiences for Incarcerated Individuals Who Identify as Transgender in a Southern Jail

**DOI:** 10.1089/trgh.2017.0046

**Published:** 2018-02-01

**Authors:** Erin McCauley, Kristen Eckstrand, Bethlehem Desta, Ben Bouvier, Brad Brockmann, Lauren Brinkley-Rubinstein

**Affiliations:** ^1^ Department of Policy Analysis and Management, College of Human Ecology, Cornell University, Ithaca, New York.; ^2^ Department of Psychiatry, University of Pittsburgh, Pittsburgh, Pennsylvania.; ^3^ Center for Prisoner Health and Human Rights, The Miriam Hospital, Providence, Rhode Island.; ^4^ Department of Social Medicine, University of North Carolina, Chapel Hill, North Carolina.; ^5^ Department of Social Medicine, Center for Health Equity Research, University of North Carolina, Chapel Hill, North Carolina.

**Keywords:** health, healthcare access, jail, transgender

## Abstract

**Purpose:** To document the health-related experiences and needs of jail detainees who self-identified as transgender women.

**Methods:** Semistructured interviews with 10 transgender women of color were conducted in a county jail in a mid-sized southern city between 2015 and 2016. Interviews were recorded and transcribed, and later analyzed using a general inductive approach.

**Results:** Participants experienced high levels of abuse and harassment, solitary confinement, mental health issues, and lack of access to hormone treatment. Participants described discrimination (both by other inmates—particularly while in special housing units—and correctional officers); harsh correctional conditions, which exacerbated mental health issues; and a marked lack of access to healthcare, including hormone treatments.

**Conclusion:** Policy changes are needed to address housing and placement issues, and to increase access to healthcare for transgender women jail detainees. Training is needed for jail staff and medical care professionals in correctional settings to better understand the unique needs and experiences of transgender people.

## Introduction

On August 6th 2016, Stacy, a 30-year-old transgender inmate in the Dade Correctional Institution in Florida City, was found dead in her cell.^1^ Stacy, who identified as female, had fought and lost to legally change her name from Justin Lee Naber to Stacy Lorraine Naber while serving a life sentence in male prison facility. Although the Florida Department of Law Enforcement declined to comment on Stacy's mental health and medical history, her family stated that she had committed suicide. The Washington Post reported that her attorney said “Naber fought for comprehensive treatment for her gender dysphoria for months but was denied each time.”^1^ Stacy was not alone in her fight for comprehensive access to healthcare as a transgender woman imprisoned in the United States. Transgender people are disproportionately incarcerated and have unique health needs that often go untreated.^2–5^

Criminal justice sites are arguably the most gender segregated of U.S. institutions,^6^ and the role of gender is complex and in flux in carceral settings. Although epidemiological data detailing the prevalence of imprisonment among transgender and gender nonconforming individuals are sparse, data suggest a much higher rate of imprisonment for transgender and gender nonconforming individuals.^5^ One in six transgender individuals have been incarcerated at some point in their lives.^7^ For black transgender people, the rates of incarceration are even higher^7^; some estimates indicate that nearly half (47%) have been incarcerated at some point.^8^

These high rates of incarceration are driven by pervasive discrimination,^5,8,9^ disproportionate poverty and homelessness,^5,8,10^ participation in street economies,^8^ and bias and abuse by law enforcement officers.^8^ History of incarceration for transgender people is associated with negative physical and mental health-related indicators.^7,11,12^ Transgender stigma is associated with reduced access to employment and healthcare, and persistent mental, and physical health issues.^13^

Before, after, and during incarceration, transgender individuals are at risk for violence and victimization and have more severe health disparities.^11^ Brown and Jones found that veterans who identify as transgender experience both significant health disparities compared with their cisgender counterparts, and were more likely to be imprisoned.^14^ Transgender and gender nonconforming people face higher rates of violence in the community,^15^ as well as during imprisonment.^7^ Transgender people experience violence early in life, are at risk of multiple types and repeated victimization, and the risk of experiencing violence remains high throughout their life.^15^ In addition, transgender people face increased risk of mistreatment and violence, economic hardship, and psychological stress, and decreased access to appropriate and adequate healthcare in the community.^16^ In addition to intersecting markers of risk, transgender individuals also encounter stigma and discrimination during incarceration.

Nearly half of transgender people who are incarcerated experience victimization during imprisonment (47%).^7^ Reports have found that between 65% and 85% of transgender detainees are put in solitary confinement, where they are isolated and lose access to recreation or visitation.^17,18^ Emmer et al.^17^ found that the use of solitary confinement for transgender individuals occurred in response to self-defense, for “protection,” or as punishment for gender expression, sexual activity, or for filing grievances. Transgender and gender nonconforming detainees in solitary confinement were also sometimes denied medical care or food as additional punishment.^17^ Solitary confinement is a traumatic experience with many deleterious mental health implications—especially for those already struggling with mental illness.^18^

During incarceration, transgender persons often lack access to healthcare services and medication.^2^ Previous studies have demonstrated that a quarter of transgender inmates report being denied access to healthcare during imprisonment,^7^ and nearly 20% of transgender individuals report being denied medical care in the community due to their gender identity.^19^ Some states allow for the continuation of hormone treatments while others do not; however, surgical treatments (e.g., gender reassignment surgery) are nearly uniformly denied.^4^

The international guidelines for the healthcare provision of transgender and gender nonconforming individuals have long emphasized the importance of both “real-life experience” (such as adopting a new gender role presentation) and the use of hormone therapies or gender-affirming surgery.^5,20^ A recent study exploring correctional healthcare providers' experiences caring for transgender detainees indicates that adequate care and gender-affirming care are not offered during imprisonment due to structural factors (such as lack of training and restrictive policies) and individual level factors (lack of cultural competence).^13^

Denying access to adequate healthcare for transgender individuals, such as hormone therapy, can cause undesired changes in appearance and is associated with depression, anxiety, and suicidality.^5,20^ This is particularly important in light of the increased risk of psychological distress and suicidality that transgender people already face. In the 2015 U.S. Transgender Survey, nearly 40% of transgender people reported experiencing serious psychological stress in the month before the survey (compared with 5% of the U.S. population broadly), 7% attempted suicide in the year before the survey (compared with 0.06% of the U.S. population broadly), and 40% had attempted suicide in their lifetime (almost nine times the U.S. rate).^16^

A review of letters from transgender inmates to the Federal Bureau of Prisons found frequent concerns around access to adequate healthcare for transgender individuals, and five lawsuits in which the department of corrections was found liable or settled.^2^ The Supreme Court cases *Estelle v. Gamble*, which ruled deliberate indifference a violation of the eighth amendment, and *Bowring v. Godwin*, which ruled psychiatric conditions a medical problem held to the standard of deliberate indifference, laid the groundwork for legal action for incarcerated transgender and gender nonconforming individuals not receiving adequate care.^5^ However, there has been an ongoing legal battle over what adequate care actually entails.^5^

Although several studies have attempted to document the state of healthcare access and healthcare policies for incarcerated transgender individuals,^4,19^ few studies have captured the lived experiences of transgender individuals' access to and experience with healthcare during incarceration.^13^ Furthermore, little is known about how treatment and healthcare access impact transgender individuals during incarceration. Of the studies that do explore transgender health issues during incarceration, many occur in the North-Eastern region of the United States, such as Pennsylvania^17^ and New York,^21^ or in California.^22^ The political and cultural climate for transgender people—in particular transgender women of color—may be markedly different in the South. This study addresses these gaps by describing the health-related experiences and needs of transgender detainees in a Southern jail system.

## Methods

We conducted 10 semistructured qualitative interviews with transgender women of color in a county men's jail in a mid-sized Southern city. Pseudonyms will be used to refer to participants. Most were incarcerated for breaking drug-related laws or for probation/parole violations. The participants reported a high number of chronic and infectious diseases. Like most other correctional facilities across the nation,^23,24^ this jail system assigned people to housing based on gender assignment at birth.

Participants were recruited through a screening question that was added to the Prison Rape Elimination Act (PREA) intake questionnaire. Any individuals who reported that they identified as transgender between the fall of 2015 and spring of 2016 were approached to participate in the study. Referrals were made by jail staff, and all 10 participants who were referred participated in the study. Participants were paid $25.00 for their participation in the form of a check that was placed in their property bag (and could be accessed upon release).

The semistructured interview guide was designed by the study principal investigators and covered the following topics: social history and previous incarceration, transgender identity development, access to healthcare in the community and correctional setting, and environmental and context issues in carceral settings that may impact health (stigma, housing, etc.). This project was approved by an Institutional Review Board and all participants provided consent before study enrollment.

Interviews were conducted by three members of the research team with training in qualitative research and experience interviewing imprisoned people from varied and diverse at-risk populations. Interviews occurred mostly in private, in the lawyer visitation room. Although no jail staff members were present, there were occasionally other inmates meeting with their lawyers in the room behind a partition. Interviews were audiorecorded and transcribed, after which the original recording was destroyed.

A general inductive approach guided the analysis of data, which allowed for the data to be formulated into themes and categories.^25^ The six coders read the transcribed data for participant responses that were similar and recurrent themes and patterns in the transcripts. Open and axial coding were then used to outline concepts among coders. Coders did a close initial coding to develop a list of possible codes. These codes were distilled into themes and subthemes for focused coding. Each theme and subtheme was assigned a code, and the codes were compiled in a codebook. Quality checks were conducted on all transcripts through iterative coding by at least two coders. Discrepancies in interpretation were resolved among the research team before final coding commenced.

## Results

Across all interviews, three primary themes emerged—issues related to (1) housing, (2) mental health, and (3) continuation of hormone medications. Housing issues that affected transgender jail detainees include special housing assignment, the use of solitary confinement, and facility preferences. Mental health issues that impacted transgender detainees include previous trauma and mental illness. Finally, the continuation of medications was a problem for most incarcerated transgender women in this study. Hereunder each theme is presented in greater detail.

### Housing

Respondents reported issues with special housing assignments, the use of segregation or solitary confinement, and highlighted their personal facility housing preferences. Housing assignments are made in these facilities based on gender assignment at birth. Participants reported being assigned or moved to “the second floor,” which is a special housing unit for people who identify as LGBT, suffer from mental illness, have committed sexual crimes, or who have disabilities. Nia said “They put me and two people who…sex offenders and stuff like that…and gay people mixed together. Which I don't think it was comfortable. I don't think that's right.” She went on to describe harassment she experienced involving a sexual offender with a mental illness in her cell. She describes her interaction with a correctional officer in reporting the event. “Even the [correctional officer], she will tell you like I told her. She like ‘he's mental’. I said, well they shouldn't [put him here], can they get him out of my room?” After Nia reported that she felt unsafe, she was still housed with the individual who had harassed her.

Another participant, Shanice, had a similar experience. She said, “I don't think it's right that they house us with rapists,” referring to the correctional staff's decision to place her on the second floor. She noted that “the rapists are the people that they, they'll try to get in the shower with me. They'll want to grab on my butt. You know, they just do all that. And when we complain about stuff like that [the jail staff] just throw it off…” Nine participants said that they felt they were not respected on the second floor, and that the second floor was stigmatizing. One participant, Raven, reported that “they watch us more,” preferring the special housing unit.

Out of the 10 participants, 4 were in solitary confinement at some point during their incarceration. Participants reported being sent to solitary confinement as punishment or for protection. Kiara reported spending 9 months and then 8 months in solitary confinement for arguing with correctional officers. She said, “it's guards, they like try to antagonize me or whatever and I tried, I got a smart mouth. So I tried to hold my peace but when they push my button, I just go off and I just get sent straight to the hole.” Solitary confinement was described as challenging to survive, and a trigger for mental health issues. Another respondent, Nia, said,

Yes, like seriously. It's hard for me to say, but it's so hard that I literally was about to hang myself. I had to start talking to God like God get me out of here. I've got court tomorrow. I'm begging. If I can't go to court I'm losing my life. You know, I was like if I can't do… I'm gonna have to go [commit suicide]. You know just, and which is not right for me to say, but it gets to that part in life that you're going to like… I have to give up, especially in here. That's what will happen.

Participants also reported that solitary confinement was used to punish behaviors that were not approved of. Raven said, “but the reason why I'm up here [in solitary confinement] is because, okay. I had a boyfriend downstairs. And we kissed. And we hugged and stuff. But they're trying to say that I gave him oral sex and we had anal sex.” Other detainees reported to the officers that she had sex. Raven said that even though there are security cameras, which could prove they never had sex, she was sent to solitary confinement. She believes she was reported because the other people on her floor were uncomfortable with being housing with a transgender person.

Participants also emphasized the complexity of facility assignment (female versus male jails) for transgender women. When asked whether she would like to be housed in a women's facility, Kayla responded,

That's a hard question to answer, if, if I had, under one condition, that I was fully transgender, but if not it will cause a problem being there too, because the women they going to want to see the penis… you know what I'm saying, that's like the men's here will want to see the breast.

Jasmin expressed similar sentiment, saying,

I would prefer to be in a facility that specializes in my gender type. Females can be just as cruel and critical as males, so that doesn't make it any easier. But for somebody who knows the lifestyle, who's experienced in the lifestyle, and we can be messy and very disorderly amongst ourselves. But, we still know the basic grounding of being transgender, and so it would be easier just to eliminate the whole, you know, big mix of confused, unconfused, crazy, sexual, whatever people, and just put a harmonious collection of [transgender] people together.

None of the participants in this study had undergone gender confirmation survey or legally changed their name. As this participant explains, not conforming with institutional definitions of gender determination can make facility assignment complex and different for each person. Some participants reported that they would rather be housed in male facilities, others would prefer female facilities, and many reported that it was a difficult choice and neither felt quite right. Angela described her identity as shifting based on context, saying “right now, I go by male, but when I'm out and I have my stuff together, I go by female.” All of the participants emphasized the importance of consulting the individual in making facility choices.

### Mental health

Participants reported struggling with mental health issues, experiencing a lack of access to adequate mental healthcare, and an increase in mental health symptoms during imprisonment. For medical services, gender determination was made based on a combination of gender assignment at birth and anatomy—causing the participants to be considered male during medical determination. Kate described being called “he” by her medical intake worker as “like a knife to your side.”

Many spoke of persistent mental health issues that pre-dated imprisonment (eight), and others spoke of how the experience of imprisonment triggered mental health struggles or complicated existing issues (seven). When asked about sexual abuse, Jasmin noted, “Oh my God. Yes, honey. I think most girls have been, and those that haven't been, you know, God bless ‘em, but yeah. I've been through a lot of abuse.’” She then went on to provide a specific example, noting, “I don't know, you know, most parents don't want a sissified son so I'd get like hit in the face with bats, drowned, put in the closet with rope, duct tape, and clothespins put all over my body, genitalia, eyes, eyelids, scrotum, all that. Handcuffed to a bed, butt naked and beaten with an extension cord. A lot of things.” Jasmin explained that she was still in contact with her parents despite this abuse, saying that as a transgender person who is in and out of incarceration she needs support, food, and housing periodically even from people who do not treat her well.

Shanice explained that the self-esteem and self-stigma issues she had overcome in her past, especially in relation to her gender identity, resurfaced during imprisonment,

It [being in jail] just takes you places. I don't know. I know this is what I want to do. This is what I've been wanting to do since I was little. It took me a while until I was grown to actually get the confidence, and you know, actually feel beautiful about myself. And, I'm in here. And it's like people are hollering at me: “Hey, look who that guy is” and “Look at, it!” I try to ignore it's only so much ignoring you can do. It still messes with you in your head. And then I don't have any support on the street so I'm in here alone. And it's just like, they're coming down on me and I don't have anyone I can talk to about that. So mentally it does do a lot. I put in a sick call and told the mental health person what I'm going through without my hormones. And she's just like, “Well, I guess that's normal. You need to just get out and get you an income so you don't come back to jail.”

Her experience of being harassed and dehumanized and being denied hormone therapy exacerbated her negative feelings of safety and self-worth. Nia reported that harassment from fellow inmates also triggered her mental health issues,

Yeah. Like, and I'm gonna be honest. Like two days and last night I felt like hanging myself. You know, I literally cried as I was reading my book because it was a little predicament, and I was like …. You hear them all day, you faggot bitch, I'm gonna kill you.

Similar to Shanice, Nia's experience of verbal abuse affected her sense of sense and in this case led to suicidal thoughts. Another participant, Alexis, reported that the experience of incarceration was affecting her mental state:

In here I can go through—my mind goes through a million things. But it is like I can't sit still, I do too many things at one, when you do too many things at one time you can't think. You can't think and you just fucked up.

These participants felt harassed by their peers, and institutional distrust and lack of institutional response limited their access to support to help deal with the harassment and isolation. The participants reported that the experience of incarceration has detrimental effects on mental health, particularly for people who are harassed or victimized during their imprisonment.

### Continuation of medication

As previously discussed, transgender individuals suffer from reduced access to healthcare in the community, which often leads to lack of treatment for known ailments and using alternative methods to seek gender-confirming treatment (such as hormone therapy).

When people are admitted to jail, they often experience a disruption in medication if they cannot provide a prescription. Alexis described an issue with accessing hormones medication, “and I talked to them about my hormones because that's what I was takin' on the street.” Jenna had been using her sister's prescription for hormone therapy while in the community due to lack of access to insurance. She reported similar difficulties getting hormone therapy while imprisoned, “I have to have a doctor and a prescription, you know, to where they can call and be like, you know, verify that I'm taking them.” In the community, lack of insurance acted as a barrier to seeking gender-confirming care, and as illustrated by these participants, many individuals access hormone medication informally. Lack of insurance, in this instance, continued to limit her access, at least in the short term, during imprisonment.

Disruption to hormone treatments can have serious implications for detainees, as Shanice described, “I was taking estrogen shots and now I'm in here having hot flashes. My hormones were going through the roof. My facial hair is slowly growing back. It's not a lot but I've been here for a week, and I can feel the peach fuzz.” She noted, “You are, basically, are stopping my process for six months because you all want to be smart. I told them you can do whatever test you have to do to see that I was taking hormones but they're not going to do that.” Shanice expressed concern about both the symptoms of withdrawal from hormone medication—such as hot flashes and physical changes, and stalling her transition process.

Seven participants reported hormone use, and six reported experiencing a disruption of medication upon intake. Two of the three participants who were not taking hormone medication before imprisonment reported that the lack of medication was due to financial reasons and lack of insurance, and expressed interest in beginning hormone therapies during imprisonment if it were an option.

## Discussion

During incarceration, the transgender women in our study experienced abuse and harassment, were often kept in solitary confinement for extended periods of time, were vulnerable to mental health issues that were exacerbated by institutional agents, and suffered from lack of access to hormone treatment primarily because they lacked a formal prescription. In addition, participants described the implications of being placed in special housing units with other stigmatized groups.

This study has important implications for both policy and practice. Housing proved to be particularly impactful and challenging for participants. Narratives highlighted the complexity of housing placement preferences and the importance of housing for participants' safety and well-being. A report by Hearts on a Wire found that the role of housing and placement was similarly important, and that being in the general population allows an opportunity to create community and reduce isolation.^17^ In contrast, being in the general population exposes transgender people to the potential of violence by other detainees, which may be overlooked or encouraged by correctional staff.^17^ However, administrative segregation and protective placements (like the use of the second floor in our study) isolate transgender individuals from support networks and increase vulnerability to assault or abuse by correctional staff.^17^ Similar to our findings, other studies have also reported high use of solitary confinement among transgender detainees.^17,18^

An analysis by Sumner and Jenness found that correctional policies that affect transgender detainees are formed by safety concerns.^26^ They argued that there are convergent policy positions across institutional sites, such as anatomy-based housing, and divergent policy positions, such as access to hormone therapies.^26^ In addition, they argued that correctional sites are concurrently gender segregation and multigendered.^26^

Owing to the complex nature of housing placements for this population and this study's participant's diverse views on housing best practices, we recommend that county jails implement an advisory board for making placement decisions for transgender and gender nonconforming people. Many different considerations must be deliberated in the placement of transgender people, including, most importantly, their own preference. The advisory board should include advocates from the transgender and gender nonconforming community, in addition to correctional officers, and other jail and medical staff. The Cook County Jail in Illinois has a “Gender Identity Disorder Committee,” which assesses the placement of transgender individuals during intake.^27^ The committee meets monthly, consists of half a dozen members (including a corrections psychologist, the chief of operations, and representation from the State Department), and considers several sources—including an interview with the incoming detainee—to make a personalized plan for housing, safety, and hormone treatment (if needed).^27^

Policy changes are needed to increase access to quality healthcare for transgender and gender nonconforming detainees. Participants in this study illustrate the challenges to continuing hormone treatment during incarceration—an issue that is under examined in the current literature. However, these findings mirror, broadly, findings related to lack of access to care for transgender individuals both in the community and in the correctional settings. Consistent with prior research showing a disruption in medication for both transgender and cisgender patients, the transgender women in this study reported being denied access to hormone treatment and many deleterious side effects of hormone treatment withdrawal. Hormone therapy disruption is associated with deleterious health effects, and hormone therapy is a medically necessary intervention for many transgender individuals.^17,20,28^ In addition, for some who identify as transgender, the disconnect between their self-identified gender and their external gender presentation can be stress producing and have negative mental health implications.^20^ This lack of access to cross-gender hormone treatment has been associated with severe negative outcomes, including suicidality and autocastration by surgical self-treatment.^29^

For several of our participants, they were unable to access hormone therapy during imprisonment because they did not have a medical prescription in the community. This is consistent with the existing literature—more than 40% of imprisoned transgender or gender nonconforming detainees who were on hormone therapies before imprisonment obtained hormones through alternative means (without a doctor prescription).^17^ Among transgender people in the community, similar lack of access to adequate health insurance and healthcare is found—a quarter of transgender people in the 2015 Transgender Survey reported trouble with their insurance related to their gender identity, more than half were denied gender-confirming surgery, a quarter were denied hormone therapy, and 33% did not access needed healthcare services due to financial barriers.^16^ Lack of access to safe and adequate healthcare in the community leads to additional barriers to healthcare while imprisoned.

The abuse and stigmatization reported by the participants in our study are not unique—Reisner et al.^7^ also found that 47% of transgender inmates experience victimization during imprisonment. A report from the University of California at Irvine found that nearly 60% of transgender inmates in a random sample reported experiencing sexual assault compared with 4.4% of the general population.^22^ Seventy-five percent of transgender inmates in the sample reported repeated sexual assault.^22^ Despite the passing and implementation of the PREA in 2003, national standards for the management of prison rape have not been universally adopted by all states.^30^ Jenness and Smyth^30^ have argued that the law remains largely symbolic. An increased risk of victimization and assault adds additional healthcare needs to the already complex and unmet healthcare needs of transgender detainees.

Transgender individuals have specific health concerns and needs, and access to trained and qualified healthcare practitioners with a deep understanding of these specific needs is imperative to protecting their health and well-being.^20^ Training for correctional and medical staff should include education related to the importance of continuing medications necessary for gender transition, and access to mental healthcare that assists transgender detainees in coping with the experience of incarceration and any possible victimization they may experience.^17^ Last, medical staff and correctional officers need to have sensitivity training to help them better understand the transgender experience, and to aid in the humanization of transgender inmates.

Several interventions have shown promise for reducing risk for transgender individuals. Stigma prevention interventions may reduce adverse health effects for transgender individuals.^13^ A transgender cultural and clinical competence intervention for healthcare providers was found to increase willingness and competency to provide appropriate healthcare for transgender individuals in the community.^13^ An intervention in Boston that used a local culturally responsive approach integrating biobehavioral techniques, health services, education, training, and advocacy also showed the promise of training and intervention when working with transgender people.^28^ And last, a study conducted in the New York City Correctional System found that LGBT training was associated with a 50% drop in patient complaints 3 months after implementation, and that a follow-up transgender healthcare policy reduced complaints to none 6 months after implementation.^21^

### Limitations

This study has several limitations, including recruitment issues, institutional barriers to conducting the interviews, and limited privacy. The research team received very few referrals for interviews during the 18 months of study enrollment. Although recruiting in correctional facilities can be challenging,^31^ in general, recruitment for this study was especially difficult. The research team had to rely on participant self-report of transgender identity—something that incarcerated individuals may be unlikely to disclose to institutional agents given the stigma attached. Therefore, our sample is likely biased. All 10 participants who were referred to us by jail staff participated in the study. However, we were reliant upon jail staff to notify us of referrals.

The small sample size poses a threat to internal validity. However, despite the small sample size, there was considerable saturation in the data in relation to key areas of interest—indicating that the threat to internal validity is minimal.^32^ In qualitative research, the number of interviews needed is directly related to the level of saturation and the nature of what the researcher seeks to explore.^32^ The population of transgender inmates who disclose their transgender status in the South may be very limited. Furthermore, Guest et al.^33^ found that saturation can be achieved after between 7 and 12 interviews—depending on the homogeneity of the sample. In addition, our key themes of housing, mental health, and healthcare access (in particularly access to hormone therapies) mirror what has found in other studies set in other locations.^34^

Interviews were conducted in the lawyer visitation area, which also has limitations. Although there was often complete privacy, at other times there were other detainees meeting with their lawyers. Owing to the sensitive nature of the interviews, the presence of other detainees may have influenced what the participants disclosed. Steps were taken to minimize this, such as sitting in the far corner with the partition between the interview and the lawyer meetings, but the threat to privacy could not always be avoided.

## Conclusion

This study has highlighted the difficulty that transgender women of color experience during imprisonment—including the overuse of solitary confinement, lack of access to hormone treatment, and reports of abuse and harassment. Our results suggest that transgender women have specific needs while imprisoned, and at the individual and institutional level, those needs often go unmet. Policy and practice reforms are needed to improve access to healthcare, housing, and treatment of transgender people, thereby improving their long-term health and well-being. This is particularly important in light of the health disparities that previously incarcerated transgender individuals experience long after imprisonment. Future research should explore how the issues that transgender women of color face in Southern jails are experienced by other groups in other contexts—such as transgender men or different geographic regions.

## Abbreviation Used

PREA: Prison Rape Elimination Act
